# Can listening-related fatigue influence well-being? Examining associations between hearing loss, fatigue, activity levels and well-being

**DOI:** 10.1080/14992027.2020.1853261

**Published:** 2021-01-04

**Authors:** Jack A. Holman, Benjamin W. Y. Hornsby, Fred H. Bess, Graham Naylor

**Affiliations:** a Hearing Sciences (Scottish Section), Division of Clinical Neuroscience, School of Medicine, University of Nottingham, Glasgow, UK; b Department of Hearing and Speech Sciences, Vanderbilt Bill Wilkerson Center, Vanderbilt University School of Medicine, Nashville, TN, USA

**Keywords:** Hearing loss, hearing devices, fatigue, well-being, activity, daily-life

## Abstract

**Objective:**

Well-being is influenced by the activities we undertake. Hearing loss may reduce well-being directly through increased listening-related fatigue due to cognitive and emotional strain in challenging situations. Hearing loss and hearing device use may also indirectly impact fatigue and well-being by altering the frequency and type of daily-life activities. This review examines the available literature to help understand the relationships.

**Design:**

We provide (i) a summary of the extant literature regarding hearing loss, hearing device use and fatigue in adults, as well as regarding fatigue and daily-life activity (work, social and physical) and (ii) a systematic search and narrative review of the relationships between hearing loss, hearing device use and activity.

**Study sample:**

The systematic search resulted in 66 eligible texts.

**Results:**

Data examining well-being in persons with hearing loss are limited. Our literature review suggests that well-being can be related directly and indirectly to hearing loss, hearing device use, activity level and listening-related fatigue.

**Conclusions:**

Variations and interactions between hearing loss, hearing device use, fatigue and activity levels can be expected to impact well-being in persons with hearing loss in direct and indirect ways. Future research linking hearing and daily-life fatigue should take account of activity levels.

## Introduction

Well-being is often described as the state of being comfortable, healthy or happy (OED Online, 2019), and is a product of each individual’s subjective feelings and beliefs (Diener 2009). Everything we do, and everything we experience has the potential to affect our well-being for better or worse (Dodge et al. 2012). Hearing loss is one of the most prevalent sensory disorders and prevalence is predicted to increase due to ageing populations (Goman, Reed, and Lin 2017). Hearing loss can have significant consequences, beyond a reduction in audibility. People with hearing loss often experience reduced well-being (Scherer and Frisina 1998; Dalton et al. 2003; Tambs 2004). There are many consequences of hearing loss which might affect well-being (Arlinger 2003). One such consequence which has recently been the topic of increased interest is that of fatigue (Hornsby, Naylor, and Bess 2016). It has long been considered that people with hearing loss may experience greater levels of fatigue in everyday life due, in part, to an increased requirement for listening effort (McGarrigle et al. 2014). While the experience of fatigue could itself be considered a symptom of reduced well-being, the impact that listening-related fatigue can have on overall well-being must also be considered.

**Figure 1.**
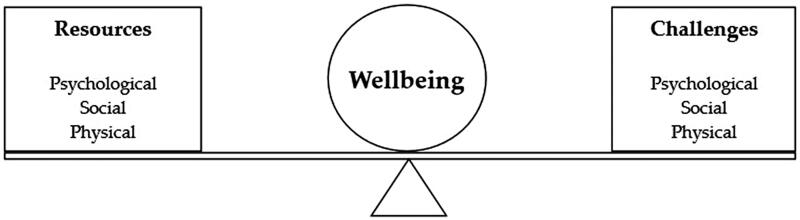
Definition of well-being as proposed by Dodge et al. (2012).

There is an unresolved debate regarding how to best define the concepts of well-being and fatigue. Dodge et al. (2012) proposed that well-being is best defined as the balance point between an individual’s resource pool and their ongoing challenges. This definition (Figure 1) identifies resources and challenges as being psychological, social and physical, demonstrating the various factors that may affect one’s perception of well-being.

The core concepts of the definition of well-being (Figure 1) are mirrored in the model of well-being proposed for use in audiology by Vercammen et al. (2020). The model suggests that the core dimensions of well-being (socio-emotional, cognitive and physical) can be influenced by hearing loss and hearing rehabilitation. To use the visual example in Figure 1, hearing loss can add socio-emotional, psychological or physical challenges, or reduce the corresponding resources, resulting in an imbalance between available resources and challenges, essentially disrupting well-being. Likewise, hearing rehabilitation could potentially provide additional resources or reduce challenges to restore balance and improve well-being in those domains.

The definition and optimal measurement of fatigue are not unanimously agreed upon, and in many cases depend on the specific area of research. Fatigue can be viewed as transient (momentary and task related) or long-term (not specifically task related) (Hornsby, Naylor, and Bess 2016). Hockey’s (2013) motivational control model of executive control, effort and fatigue depicts fatigue as a mechanism which prompts the reassessment of motivational priorities and the utility of alternative actions. Motivation in any given situation is partly dependent on the control a person has, as well as intrinsic and extrinsic factors such as enjoyment and satisfaction or duty (Schneider et al. 2019). Additionally, it has been argued that fatigue is a multi-dimensional construct. Mental fatigue, physical fatigue, emotional fatigue and vigour/vitality have been identified as separate dimensions of fatigue (Stein et al. 2004). However, others argue that while people suffering from fatigue may have diverse experiences, these all reflect a single underlying latent construct (Michielsen et al. 2004).

**Figure 2.**
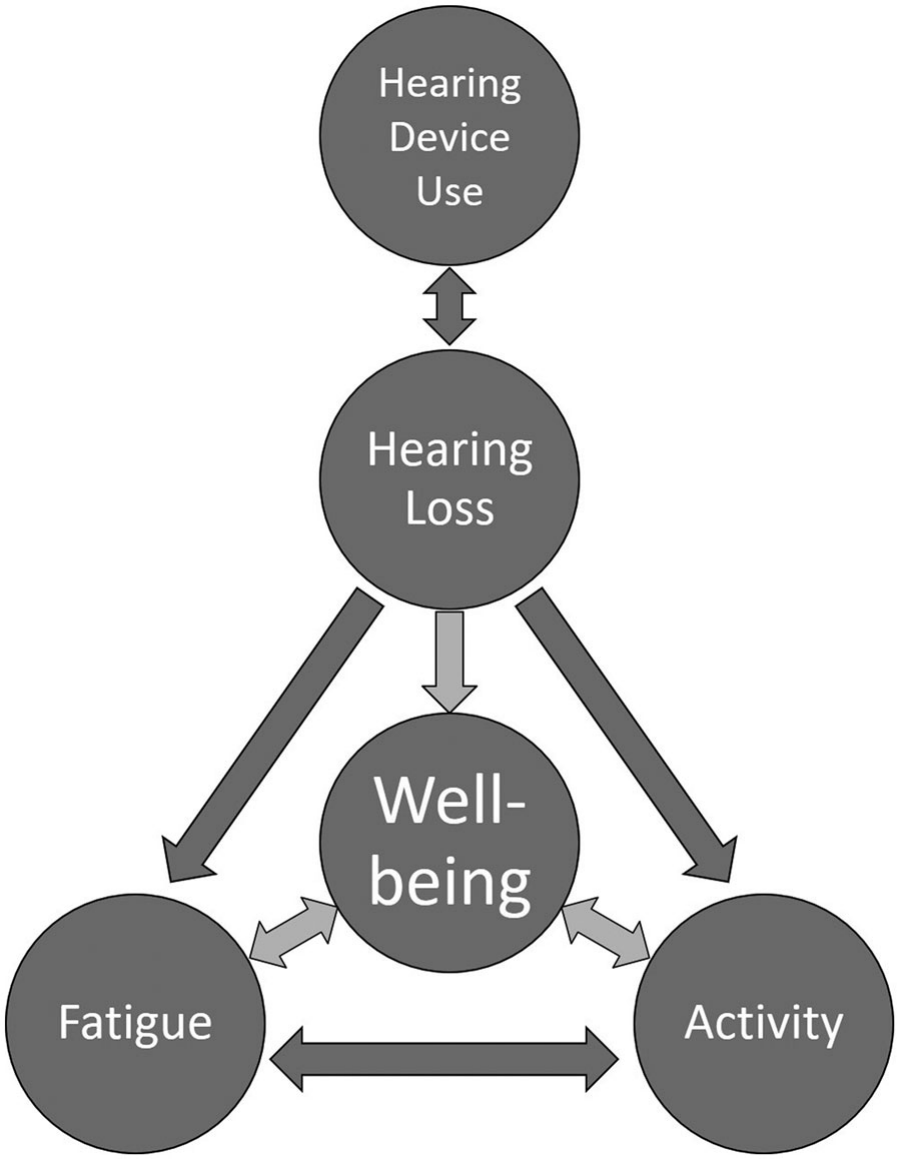
A theoretical framework of associations between hearing loss, hearing device use, listening-related fatigue, activity and individual well-being.

The relationship between fatigue and well-being is not well understood but appears complex and is likely not unidirectional (Figure 2). Severe fatigue may simply be a symptom of poor well-being due to other factors. Negative emotional factors such as depression have been linked to both fatigue and well-being (Beekman et al. 2002; Lavidor, Weller, and Babkoff 2002). Alternatively, experiencing severe fatigue on a regular basis could also have a direct causal effect, leading to a reduction in well-being. Fatigue could manifest as a socio-emotional, cognitive or physical challenge to an individual, thus affecting overall well-being (Haack and Mullington 2005; Smith 2018). It has been proposed that listening-related fatigue in people with hearing-loss occurs through the direct impact of hearing loss on audibility and auditory processing, and the subsequent increase in required listening effort, in given listening situations (McGarrigle et al. 2014; Hornsby, Naylor, and Bess 2016).

In addition, hearing loss may have an indirect impact on fatigue by affecting a person’s activity level. This activity mediated change in fatigue could in turn influence well-being (Figure 2). For example, it is feasible that psychosocial difficulties that arise due to hearing loss (Heffernan et al. 2016) could lead to an alteration (most likely a reduction) of a person’s level of activity at work, in social settings or general physical activity. As challenging situations themselves likely impact feelings of fatigue, a change in daily activity levels could in turn lead to a shift in longer-term fatigue. Daily-life activity can be considered as an integral component of well-being in terms of both resources and challenges. One’s level of physical and social activity has been shown to be directly linked to well-being, as has the degree of satisfaction with one’s level of work activity (Burke and Greenglass 2000; McAuley et al. 2000; Netz et al. 2005). The directionality of the link is not addressed in these studies, however both directions of causality are equally feasible. To date, the relationship between listening-related fatigue and activity level not been investigated. This paper provides an initial examination of these relationships, as a first step towards understanding their association with well-being.

This review addresses potential interactions between hearing loss/hearing device use, fatigue, activity and well-being (Figure 2) by first providing a summary of the available literature regarding the impact of hearing loss and hearing device fitting on fatigue (1). Following this, we assess the well-documented relationship between activity levels and fatigue (2). Finally, we present a systematic search and narrative review of the literature pertaining to the relationships between both hearing loss and hearing device use, and activity levels (3). By investigating the role of activity level on listening related fatigue we hope to more fully assess its direct and indirect impact on well-being.

## Hearing loss, hearing device use and fatigue

### Hearing loss and fatigue

Qualitative studies suggest that people with a hearing loss experience fatigue as a result of additional difficulty in listening situations (Hetu et al. 1988; Holman et al. 2019). While not a universal finding, multiple studies suggest that people with hearing loss experience more fatigue than people without hearing loss (Holman, Drummond, and Naylor 2020). Most relevant studies have used subjective measures to investigate predominantly long-term fatigue. Of those, a majority of results support the hypothesis that hearing loss is linked to increased levels of fatigue (Grimby and Ringdahl 2000; Ringdahl and Grimby 2000; Karinen et al. 2001; Dalton et al. 2003; Cheng, Gurland, and Maurer 2008; Nachtegaal et al. 2009; Jahncke and Halin 2012; Fredriksson et al. 2016; Alhanbali et al. 2017; Svinndal et al. 2018). However, others have shown mixed (Hornsby and Kipp 2016; Alhanbali et al. 2018; Dwyer et al. 2019) or non-significant results (Wagner-Hartl and Kallus 2018; Wang et al. 2018). Some studies have used physiological measures such as cortisol levels, pupil dilation and auditory event-related potentials to investigate the impact of hearing loss on measures associated with fatigue (Bess et al. 2016; Gustafson et al. 2018; Wang et al. 2018; Dwyer et al. 2019). However, as these techniques measure related but separate physiological responses such as stress and arousal, more refinement of these experimental techniques is necessary before definitive conclusions regarding fatigue can be drawn. For example, recent work has successfully measured fatigue using pupillometry in adults and children without hearing loss, which could lead to its use in the future in those with hearing loss (McGarrigle et al. 2017a, 2017b).

### Hearing device use and fatigue

Qualitative studies have shown that hearing device use can result in increased or decreased levels of fatigue (Holman et al. 2019; Davis et al. 2020, in review). After hearing device fitting, the benefits of device use in any given situation may be counteracted by an increase in the variety and duration of conversational situations entered into, assuming related factors such as motivation and control remain unchanged. Only six studies investigating the relationship between hearing device use and subjective fatigue have been identified in a recent review on this topic (Holman, Drummond, and Naylor 2020). Results from that review are summarised here. All studies except one (Hornsby 2013) investigated long-term fatigue. Whether a person owns a hearing device or not is often the metric used by studies, where actual intensity of use is rarely measured. Three prospective non-randomised control trials used self-report questionnaires to show that the provision of a first, or a second, cochlear implant reduced fatigue (Chung et al. 2012; Harkonen et al. 2015b, 2015a). The evidence regarding the benefits of acoustic hearing aid use on self-reported fatigue is less consistent. One study using self-report questionnaires found significantly less fatigue in people who wear acoustic hearing aids compared to people with a hearing loss who do not (Bisgaard and Ruf 2017). Two other studies found no significant difference between groups (Hornsby 2013; Alhanbali et al. 2017). However, in a crossover study using a dual task paradigm, Hornsby (2013) did find a significant transient fatigue-related objective benefit from hearing aid use. Specifically, hearing aid use appeared to mitigate some fatigue-related effects on sustained attention as measured using a dual-task reaction time paradigm. At the same time, recent qualitative research suggests that for some adults with hearing loss, active listening with hearing devices can, itself, be a fatiguing activity. These adults may remove or turn off their hearing device to take “listening breaks” to reduce, or prevent the development of, listening-related fatigue (Davis et al. 2020, in review).

The ability to generalise the findings from these studies is hindered by their small number, and by the wide variety of study designs. Additionally, cross-sectional studies often do not consider that hearing device ownership does not equate to hearing device use. Despite this, the hypothesis that hearing device usage can, under some conditions, reduce fatigue does seem to have merit and warrants further investigation.

## Activity and fatigue

Recall that hearing loss may be linked directly and indirectly to fatigue and well-being. Hearing loss can directly influence fatigue through increased requirement for listening effort. Hearing loss could also indirectly affect fatigue by changing the level of daily-life activity an individual undertakes, and potentially affect activity by changing the individual’s fatigue level. In this section we investigate part of this indirect link through a review of the literature connecting the level of daily-life activity (work, social and physical) with fatigue.

### Work activity and fatigue

Work activity can be viewed as either work status (i.e. in work versus unemployed) or the level of work activity such as the number of hours worked. With regard to the effect of the level of work activity on fatigue, evidence shows that high levels of work activity are linked to higher fatigue (Ono et al. 1991; Park et al. 2001; Nagashima et al. 2007). The relationship between unemployment and fatigue is dependent on the control a person has over their unemployment. Retiring from work has been linked to reduced physical and mental fatigue (Westerlund et al. 2010). In contrast, the financial and social difficulties associated with unwanted unemployment and job-seeking have been shown to be linked to increased fatigue (Lim et al. 2016). This introduces the recurring theme of the relationship between activity and fatigue not being monotonic. In this instance, an important factor seems to be a person’s satisfaction with their work status.

### Social activity and fatigue

Regarding the impact of social activity on fatigue, a key factor is the enjoyment of the social activity and the motivation to do it. When there is intrinsic motivation, or when the activity is enjoyed, social activity may lead to a reduction in fatigue and beneficially influence recovery with regard to energy (Oerlemans, Bakker, and Demerouti 2014; Oerlemans and Bakker 2014; Ten Brummelhuis and Trougakos 2014). Related studies have also shown that feelings of fatigue affect our participation in various social activities. Agahi and Parker (2005) found that older adults with higher self-reported levels of fatigue were less likely to engage in leisure activities. Another study using ecological momentary assessment found that people with higher fatigue scores on a given day were more likely to be at home over the next two days, and therefore less likely to be socialising (Ravesloot et al. 2016). This evidence suggests that social situations which are not enjoyed or intrinsically motivated could lead to fatigue, which in turn could make the person less likely to seek out future social activities. If the avoided social activities are only those which are not enjoyable, then further fatigue could be avoided. However, if potentially enjoyable social activities are also avoided, then the person would not experience the positive benefits regarding energy recovery and lower fatigue.

### Physical activity and fatigue

Numerous studies have examined the effects of physical activity on fatigue. The research is primarily divided between investigations into the effect of overtraining in athletes, and the effect of exercise in healthy or unhealthy populations. Here, the latter was of primary interest. This area is often studied by examining the effects of exercise interventions on energy and fatigue. In healthy but sedentary populations, chronic exercise (repeated sessions of exercise over a period of time) has been identified in several studies as increasing feelings of energy and reducing fatigue (Jette et al. 1996; Annesi 2002; Puetz, Flowers, and O'Connor 2008). However, this conclusion has not been reached by all studies, which may be due to initially high levels of energy and low levels of fatigue in some individuals (O'Connor and Puetz 2005). More conclusive benefits in terms of reduced fatigue have been identified when exercise has been used as an intervention for people with various fatiguing medical conditions (Dimeo et al. 1999; Quittan et al. 1999). On the other hand, experimentally induced physical inactivity has also increased ratings of fatigue (Mondin et al. 1996; Ishizaki et al. 2002). We thus find that the relationship between physical activity and fatigue is not montonic; fatigue is highest at both extremes of physical activity.

## Hearing loss, hearing device use and activity levels

The evidence above highlights that a change in activity level may affect fatigue. Therefore, before concluding that hearing loss and/or hearing device use affect fatigue, it is necessary to investigate whether hearing loss and subsequent hearing device use might also alter a person’s daily activity levels. To this end, we conducted a review of the available evidence.

For the purposes of this review, two focussed questions were used as the basis for determining literature search terms. (Q1) Is there a relationship between hearing loss and work, social or physical activity level? (Q2) Is there a relationship between hearing device use and work, social or physical activity level? Given the generally negative consequences of hearing loss and positive consequences of hearing device use, one would expect that hearing loss would be related to decreased work (referred to in the following as hypothesis H1_W), social (H1_S) and physical activity (H1_P), and that hearing device use would be related to increased work (H2_W), social (H2_S) and physical activity (H2_P). These six hypotheses were used as the basis for documenting the review.

## Methods

### Search strategy

Systematic searches were conducted in five bibliographic databases: Embase, MedLine, Web of Science, Psychinfo and the Cochrane Library. The search variables used included control terms and free text terms. All English language peer reviewed research articles were included initially from inception until 1 February 2017. An updated search included all studies until 14 May 2020. The full search terms can be found in supplemental digital content 1.

### Inclusion and exclusion

In order to determine which studies to include in the review, the Population, Intervention, Control, Outcomes and Study design strategy (PICOS) was utilised. The PICOS strategy is widely used in order to identify relevant studies for inclusion in reviews.

The population was adults (>18 years old) with a hearing loss. The exposure variable of interest (“Intervention” in the PICOS framework) for (Q1) was hearing loss (presence or severity of hearing loss, either self-reported or measured objectively), and for (Q2) was hearing device use (0 vs any, or 1 vs 2 hearing devices). All possible hearing devices were considered, although these were predominantly hearing aids and cochlear implants. The control was a population without the corresponding “intervention” of either hearing loss or hearing device, measured by within group comparison, between group comparison or within-subjects repeated measures design. The primary outcomes were work, social and physical activity. This was determined in terms of the quantity of each activity, rather than dysfunction, ability in or quality of the activity. Potential activity and qualitative aspects of activity such as enjoyment are all relevant and important aspects of the broader construct of activity. However, for this review we chose to focus on activity level because of its previously described link to fatigue and well-being. This review provides a starting point from which future research can build. Measures of activity level could be subjective or objective, measured over any timescale. Randomised controlled trials, non-randomised controlled trials, experimental studies with repeated measures design and observational studies were included. Qualitative studies were excluded from the review, as level/quantity of activity was of primary interest.

After removing duplicates from the list of all articles identified from the searches of the databases, author JH screened the titles and abstracts for potential relevance. Once narrowed down, the reference lists of relevant studies were explored for additional relevant studies. Authors JH and GN then independently examined the full texts of the remaining potential studies. Studies were categorised as either “yes” where inclusion was certain, “maybe” when there was doubt, or “no” when the study did not meet the requirements for inclusion. The lists from each researcher were then compared, and any discrepancies were discussed and resolved.

## Results

### Search output

From the initial database searches, after removal of duplicates, 2977 studies were retrieved. Based on inspection of titles and abstracts this was then narrowed down to 127 studies. Through inspection and discussion between authors JH and GN, this was narrowed further to 58 studies. The reference lists of those selected studies were examined for potentially relevant literature. After an updated search in May 2020, eight further relevant studies were identified. Thus, a final total of 66 studies were identified to answer the two focussed research questions (Tables 2 and 3 for full details). Of these, three studies addressed both questions (Lee, Gomez-Marin, and Lee 1996; Pryce and Gooberman-Hill 2012; Christensen and Datta Gupta 2017). Three studies provided results for two different types of activity each (Bess et al. 1989; Fisher et al. 2015; Lee and Noh 2015). The results of each study were scrutinised in order to determine whether the evidence supported the review hypotheses. Each result was assigned either “+” if the result supported the hypothesis, “=” if no effect was found or “–” if the result refuted the hypothesis. Most of the studies reviewed did not discuss causality or suggest any directionality in the relationships examined, but those which did are mentioned in the results below. No formal assessment of evidence quality was made as it was not deemed sufficiently beneficial for this style of review given the volume of information being discussed. However, we do describe important characteristics of the identified studies including number of participants, method of intervention measurement and method of outcome measurement (Tables 2 and 3).

**Table 2.** Identified studies relating to Q1: Does hearing loss have an effect on work, social or physical activity level?.

#	Author (year)	Intervention	Activity type	Number of participants	Measurement of hearing loss	Measurement of activity	Findings (one sign per result)
1	Bess et al. (1989)	B	Social	153	PTA (average)	SR Questionnaire (V)	+
		B	Work	153	PTA (average)	SR Questionnaire (V)	=
2	Brink and Stones (2007)	A	Social	12,254	Assessed by investigator	IR Questionnaire (V)	+
3	Bullis et al. (1995)	A	Work	439	Specialist group	Employment status, SR Questionnaire (NV)	+
4	Chan et al. (2019)	A	Physical	3790	SR Questionnaire (NV)	SR Questionnaire (V)	+
5	Choi et al. (2016)	A	Physical	1669	PTA (average) & SR Questionnaire (NV)	Accelerometer	+ =
6	Christensen and Datta Gupta (2017)	A	Work	2407	PTA (average) & Questionnaire (NV)	Disability benefits through hearing loss, SR Questionnaire (NV)	+
7	Clark, Bond, and Sanchez (1999)	A	Social	1052	PTA (average)	SR Questionnaire (V)	=
8	Crews and Campbell (2004)	A	Social	9447	SR Questionnaire (NV)	SR Questionnaire (NV)	+
9	Curhan et al. (2013)	A	Physical	68,421	SR Questionnaire (NV)	SR Questionnaire (NV)	+
10	Emmett and Francis (2015)	A	Work	3379	PTA (average)	Employment status, SR Questionnaire (NV)	+
11	Engdahl et al. (2015)	A	Physical	31,547	PTA (average)	SR Questionnaire (NV)	+
12	Fischer et al. (2014)	A	Work	3753	PTA (average)	Retirement, SR Questionnaire (NV)	=
13	Garramiola-Bilbao and Rodriguez-Alvarez (2016)	A	Work	1224	Questionnaire (NV)	Employment status, SR Questionnaire (NV)	+
14	Gispen et al. (2014)	A	Physical	706	PTA (average)	Accelerometer & SR Questionnaire (NV)	+ +
15	Haas et al. (2016)	A	Physical	1221	PTA (average)	SR Questionnaire (V)	+
16	Hasson et al. (2010)	A	Work	18,734	SR Questionnaire (NV)	Employment status, SR Questionnaire (NV)	+
17	Helvik, Krokstad, and Tambs (2013)	A	Work	25,740	PTA (average)	Early retirement, SR Questionnaire (NV)	+
18	Hogan et al. (2009)	A	Work	43,233	SR Questionnaire (NV)	Employment status, SR Questionnaire (NV)	+
19	Joo, Han, and Park (2015)	A	Physical	11,266	PTA (average)	SR Questionnaire (V)	+
20	Jung and Bhattacharyya (2012)	A	Work	∼40,000	Medical records	Employment status, SR Questionnaire (NV)	+
21	Koyanagi, Stubbs, and Vancampfort (2018)	A	Physical	206,356	Assessed by investigator	SR Questionnaire (V)	+
22	Kramer, Kapteyn, and Houtgast (2006)	A	Work	210	Specialist group	Sick leave due to distress, SR Questionnaire (V)	+
23	Lampropoulou (1992)	A	Work	148	Specialist group	Employment status, SR Questionnaire (NV)	+
24	Lee, Gomez-Marin, and Lee (1996)	A	Work	2694	PTA (average)	Employment status, SR Questionnaire (NV)	+
25	Liljas et al. (2016)	A	Social	3981	SR Questionnaire (NV)	SR Questionnaire (NV)	+
26	Linssen et al. (2014)	B (longitudinal)	Physical	1823	PTA (average)	SR Questionnaire (NV)	=
27	Loprinzi et al. (2013)	A	Physical	1485	PTA (average)	Accelerometer	=
28	Loprinzi et al. (2013)	A	Physical	682	PTA (average)	Accelerometer	+
29	Mick et al. (2018)	A	Social	21,241	SR Questionnaire (NV)	SR Questionnaire (NV)	=
30	Mikkola et al. (2015)	A	Social	848	SR Questionnaire (NV)	SR Questionnaire (NV)	+
31	Mikkola et al. (2016)	A	Social	524	SR Questionnaire (NV)	7-day activity diary	+
32	Norris and Cunningham (1981)	A	Social	50	PTA	SR Questionnaire (NV)	=
33	O'Neill et al. (1988)	A	Social	12	PTA (average)	SR Questionnaire (V)	=
34	Park et al. (2016)	A	Work	1628	PTA (average)	Hours worked, SR Questionnaire (NV)	–
35	Parving and Christensen (1993)	A	Work	218	PTA (one frequency)	Employment status, SR Questionnaire (NV)	+
36	Polku et al. (2015)	A	Physical	848	SR Questionnaire (NV)	SR Questionnaire (V)	+
37	Pryce and Gooberman-Hill (2012)	A	Social	18	SR Questionnaire (NV)	SR Questionnaire (NV)	+
38	Resnick, Fries, and Verbrugge (1997)	A	Social	18,873	SR Questionnaire (NV)	SR Questionnaire (NV)	+
39	Rydberg, Gellerstedt, and Danermark (2010)	A	Work	2144	Specialist group	Employment status, SR Questionnaire (NV)	+
40	Schroedel and Geyer (2000)	A	Work	240	Specialist group	Employment status, SR Questionnaire (NV)	=
41	Shukla et al. (2019)	A	Social	12,311	SR Questionnaire (NV)	SR Questionnaire (NV)	+
42	Souza, Fillenbaum, and Blay (2015)	A	Physical	6924	SR Questionnaire (NV)	SR Questionnaire (NV)	=
43	Stam et al. (2013)	A	Work	1888	Speech in noise test	Employment status, SR Questionnaire (NV)	+
44	Svinndal et al. (2018)	A	Work	3330	SR Questionnaire (NV)	Employment status, SR Questionnaire (NV)	+ =
45	Tehranchi and Jeyakumar (2020)	A	Work	∼3.5 mil	SR Questionnaire (NV)	Employment status, SR Questionnaire (NV)	+
46	Vesterager, Salomon, and Jagd (1988)	A	Physical	71	Specialist group	SR Questionnaire (NV)	=
47	Viljanen et al. (2014)	A	Social	27,536	SR Questionnaire (NV)	SR Questionnaire (NV)	=
48	Woodcock and Pole (2008)	A	Work	131,535	SR Questionnaire (NV)	Employment status, SR Questionnaire (NV)	+
49	Yamada et al. (2012)	A	Social	1254	SR Questionnaire (NV)	SR Questionnaire (NV)	=

A: HL vs NH; B: Level of HL; PTA: Pure tone audiometry (averaged across frequencies, or below threshold in one frequency); Specialist group: participants recruited as they are part of a group solely for people with a hearing loss; SR: Self-report; IR: Investigator-report; V: Validated questionnaire; NV: Non-validated questionnaire, or researchers unable to ascertain validity.

“+” result supported the hypothesis, “=” result did not support the hypothesis, “–” result refuted the hypothesis.

**Table 3.** Identified studies relating to Q2: Does hearing device use have an effect on work, social or physical activity level?

#	Author (year)	Intervention	Activity type	Number of participants	Measurement of activity	Findings (one sign per result)
1	Chee et al. (2004)	B (one group post fitting)	Work	30	Employment status, SR Questionnaire (NV)	+
2	Christensen and Datta Gupta (2017)	A1 (assistive devices)	Work	2407	Disability benefits through hearing loss, SR Questionnaire (NV)	+
3	Clinkard et al. (2015)	B3	Work	65	Employment status, SR Questionnaire (NV)	+
4	Dawes et al. (2015)	A1	Social	3753	SR Questionnaire (NV)	=
5	Farinetti et al. (2015)	AB: With or without contralateral HA	Social	116	SR Questionnaire (V)	+
6	Fazel and Gray (2007)	B3	Work	65	Employment status, SR Questionnaire (NV)	+
7	Fisher et al. (2015)	A1	Physical	5172	SR Questionnaire (NV)	+
		A1	Social	5172	SR Questionnaire (NV)	+
8	Fuentes-Lopez et al. (2017)	A1	Social	4766	SR Questionnaire (NV)	=
9	Hogan et al. (2001)	B1	Social	202	SR Questionnaire (V)	+
10	Kos et al. (2007)	B3	Work	67	Employment status, SR Questionnaire (NV)	=
11	Lee and Noh (2015)	A3 (uptake of HAs)	Work	119	Employment status, SR Questionnaire (NV)	+
		A3 (uptake of HAs)	Social	119	SR Questionnaire (NV)	+
12	Lee, Gomez-Marin, and Lee (1996)	A1	Work	2694	Employment status, SR Questionnaire (NV)	=
13	Meister et al. (2005)	A (benefit of HA)	Social	150	SR Questionnaire (NV)	+
14	Pryce and Gooberman-Hill (2012)	A1	Social	18	SR Questionnaire (NV)	=
15	Rafferty et al. (2013)	B3	Social	80	SR Questionnaire (NV)	+
16	Sawyer et al. (2019)	A1	Social	18,730	SR Questionnaire (NV)	+
17	Simpson et al. (2019)	A1	Work	857	Retirement status, SR Questionnaire (NV)	=
18	Stephens, Vetter, and Lewis (2003)	A1	Physical	66	SR Questionnaire (NV)	=
19	Tesch-Romer (1997)	A1	Social	148	SR Questionnaire (V)	=
20	Winn (2006)	A1 (amount of time HA worn)	Work	60	Employment status, SR Questionnaire (NV)	=

A: Hearing aid; B: Cochlear implant; 1: 0 vs any; 2: 1 vs 2; 3: Repeated measures; SR: Self-report; V: Validated questionnaire; NV: Non-validated questionnaire, or researchers unable to ascertain validity.

“+” result supported the hypothesis, “=” result did not support the hypothesis, “–” result refuted the hypothesis

The consolidated results and support for hypotheses are shown in Table 1. In the following sections, these results are discussed in detail for each of the six hypotheses.

**Table 1.** Results and support for hypotheses from identified studies.

	Work activity			Social activity			Physical activity		
	+	=	–	+	=	–	+	=	–
Hearing Loss (decreased activity)	17	4 H1_W	1	9	6 H1_S	0	10	5 H1_P	0
Hearing Devices (increased activity)	5	4 H2_W	0	7	4 H2_S	0	1	1 H2_P	0

“+” result supported the hypothesis, “=” result did not support the hypothesis, “–” result refuted the hypothesis.

Some studies produced more than one result, hence summations in this table do not always equate to the number of studies.

#### H1_W – Hearing loss and work activity

Twenty-one studies were identified which addressed H1_W, that hearing loss is associated with decreased work activity. In general, the weight of evidence supported the hypothesis, with 17 studies reporting results that supported H1_W (Lampropoulou 1992; Parving and Christensen 1993; Bullis et al. 1995; Lee, Gomez-Marin, and Lee 1996; Kramer, Kapteyn, and Houtgast 2006; Woodcock and Pole 2008; Hogan et al. 2009; Hasson et al. 2010; Rydberg, Gellerstedt, and Danermark 2010; Jung and Bhattacharyya 2012; Helvik, Krokstad, and Tambs 2013; Stam et al. 2013; Emmett and Francis 2015; Garramiola-Bilbao and Rodriguez-Alvarez 2016; Tehranchi and Jeyakumar 2020). Five studies reported results that showed either no effect or a negative effect (Bess et al. 1989; Schroedel and Geyer 2000; Fischer et al. 2014; Park et al. 2016). Svinndal et al. (2018) found a significant result for women but not for men, and therefore provide one result supporting H1_W, and one not supporting H1_W.

In most cases the population under investigation was working age adults, however three studies measured older populations (Bess et al. 1989; Helvik, Krokstad, and Tambs 2013; Fischer et al. 2014) and two measured younger adults specifically (Parving and Christensen 1993; Bullis et al. 1995). All identified studies investigated the presence of a hearing loss (i.e. differences in activity level between those with a hearing loss and those without) apart from Bess et al. (1989) who predominantly investigated health status as a function of progressive hearing impairment. Most studies investigated employment level as being either employed or unemployed. However, three studies investigated the relationship between hearing loss and early retirement (Helvik, Krokstad, and Tambs 2013; Fischer et al. 2014; Christensen and Datta Gupta 2017), and three studies measured work activity as the number of hours worked (Kramer, Kapteyn, and Houtgast 2006; Stam et al. 2013; Park et al. 2016).

#### H1_S – Hearing loss and social activity

Fifteen studies were identified which addressed H1_S, that hearing loss is associated with decreased social activity. The available evidence gave equivocal support for H1_S. Of the 15 studies, 9 supported the hypothesis (Bess et al. 1989; Resnick, Fries, and Verbrugge 1997; Crews and Campbell 2004; Brink and Stones 2007; Pryce and Gooberman-Hill 2012; Mikkola et al. 2015; Liljas et al. 2016; Mikkola et al. 2016; Shukla et al. 2019), while six found no effect (Norris and Cunningham 1981; O'Neill, Brandy, and Deck 1988; Clark, Bond, and Sanchez 1999; Yamada et al. 2012; Viljanen et al. 2014; Mick et al. 2018).

The review sought to find studies that measured the level of activity, as opposed to the potential ability, dysfunction, or willingness to participate, in activity. Some of the studies reviewed, used surveys which intermingled questions targeting social activity level with others that were less relevant. Despite this limitation, in light of the limited available evidence, those studies whose outcome measures only partly measured activity level were kept in the review.

The populations under investigation in the identified studies were usually elderly. Only two studies did not investigate the elderly specifically (Mick et al. 2018; Shukla et al. 2019), yet their samples were still largely elderly (age range 45–70 with 47% >60 years old; 84% >65 years old respectively). No studies using young populations were found. All studies compared social activity level in people with a hearing loss versus people without a hearing loss. All studies used questionnaires to assess social activity. While most studies calculated the self-reported number of social activities participated in over a period of time, others analysed related factors. People with a hearing loss were found to spend shorter durations in social activities (Brink and Stones 2007), and also spend less time out of the home where social activities could occur (Mikkola et al. 2016).

#### H1_P – Hearing loss and physical activity

Fourteen studies addressed H1_P, that hearing loss is associated with decreased physical activity. In general, the weight of evidence gave moderate support to H1_P, with ten studies reporting confirmatory results (Curhan et al. 2013; Loprinzi 2013; Gispen et al. 2014; Engdahl et al. 2015; Joo, Han, and Park 2015; Polku et al. 2015; Choi et al. 2016; Haas et al. 2016; Koyanagi, Stubbs, and Vancampfort 2018; Chan et al. 2019) and five reporting no effect (Vesterager, Salomon, and Jagd 1988; Loprinzi et al. 2013; Linssen et al. 2014; Souza, Fillenbaum, and Blay 2015; Choi et al. 2016). Two studies are listed as reporting two findings (Gispen et al. 2014; Choi et al. 2016). The method of measurement of hearing loss and physical activity in certain included studies meant that some results were less reliable than others (Table 2).

Support for the hypothesis that hearing loss may be related to physical activity level came from several studies using objective and subjective measures, in different populations (e.g. older and younger age groups). The population under investigation was elderly in four studies (Polku et al. 2015; Souza, Fillenbaum, and Blay 2015; Choi et al. 2016; Chan et al. 2019). All studies investigated the presence of a hearing loss apart from one study which investigated the deterioration of hearing ability over time (Linssen et al. 2014). Eight studies using subjective measures of physical activity provided results that supported H1_P (Curhan et al. 2013; Gispen et al. 2014; Engdahl et al. 2015; Joo, Han, and Park 2015; Polku et al. 2015; Haas et al. 2016; Koyanagi, Stubbs, and Vancampfort 2018; Chan et al. 2019). Four studies using subjective measures of physical activity provided results which found no effect (Vesterager, Salomon, and Jagd 1988; Linssen et al. 2014; Souza, Fillenbaum, and Blay 2015; Choi et al. 2016). Four studies used accelerometers as an objective measure of physical activity. Three results supported H1_P (Loprinzi 2013; Gispen et al. 2014; Choi et al. 2016), one was non-significant (Loprinzi et al. 2013).

#### H2_W – Hearing devices and work activity

A total of nine studies addressed the hypothesis (H2_W) that hearing device use is related to increased work activity: five supported the hypothesis (Chee et al. 2004; Fazel and Gray 2007; Clinkard et al. 2015; Lee and Noh 2015; Christensen and Datta Gupta 2017) and four reported no effect (Lee, Gomez-Marin, and Lee 1996; Winn 2006; Kos et al. 2007; Simpson et al. 2019). Five studies investigated hearing aid use and four investigated cochlear implants. Although a majority of findings supported the hypothesis, this was primarily dependent on the population (hearing aid or cochlear implant users) under investigation.

Three relevant studies which investigated cochlear implant use supported the hypothesis (Chee et al. 2004; Fazel and Gray 2007; Clinkard et al. 2015), and one did not (Kos et al. 2007). All four studies measured employment status after implantation compared to before using subjective questionnaires, which unlike the other five studies suggests a possible causal relationship. Three studies were repeated measures designs, whereas Chee et al. (2004) sampled a group of cochlear implant wearers once post-implantation.

Two studies which investigated hearing aid use supported the hypothesis (Lee and Noh 2015; Christensen and Datta Gupta 2017), but three studies found no effect (Lee, Gomez-Marin, and Lee 1996; Winn 2006; Simpson et al. 2019). Christensen and Datta Gupta (2017) investigated the impact of assistive devices in the workplace which often, but do not always, rely on hearing aid use. Of the other studies, two investigated hearing aid uptake (Lee and Noh 2015; Simpson et al. 2019), one investigated those who use hearing aids against those who do not (Lee, Gomez-Marin, and Lee 1996), and another investigated the amount of time hearing aids are used (Winn 2006). All studies measured self-reported outcomes. The outcome of interest was employment status in three studies (Lee, Gomez-Marin, and Lee 1996; Winn 2006; Lee and Noh 2015), with one study measuring retirement (Simpson et al. 2019) and one measuring leaving the workforce through claims for disability benefits (Christensen and Datta Gupta 2017).

#### H2_S – Hearing devices and social activity

The literature shows some support for the hypothesis that hearing device use is related to increased social activity (H2_S). Eleven studies addressed this issue and seven of these studies supported the hypothesis (Hogan et al. 2001; Meister et al. 2005; Rafferty et al. 2013; Farinetti et al. 2015; Fisher et al. 2015; Lee and Noh 2015; Sawyer et al. 2019). Four studies reported no significant relationship between device usage and social activity (Tesch-Romer 1997; Pryce and Gooberman-Hill 2012; Dawes et al. 2015; Fuentes-Lopez et al. 2017). None suggested hearing devices are related to decreased social activity.

Eight of the 11 studies included in the review investigated hearing aid use, while two studies investigated cochlear implants (Hogan et al. 2001; Rafferty et al. 2013), and Farinetti et al. (2015) investigated the benefits of a cochlear implant used in conjunction with a hearing aid or without a hearing aid. The population under investigation was elderly in five studies (Tesch-Romer 1997; Pryce and Gooberman-Hill 2012; Dawes et al. 2015; Fisher et al. 2015; Fuentes-Lopez et al. 2017). Most studies investigated participants with versus without hearing devices. One study investigated the uptake and successful use of a hearing aid (Lee and Noh 2015) and another investigated the benefit received from hearing aid use (Meister et al. 2005). All studies measured social activity level using self-report questionnaires.

#### H2_P – Hearing devices and physical activity

Only two studies were identified which addressed the hypothesis that hearing device use is related to increased physical activity (H2_P). Both studies investigated elderly populations. Stephens, Vetter, and Lewis (2003) found no significant relationship between hearing device use and self-reported activities including physical activity, however the result was stated in the narrative of the study, not as a formal result. Fisher et al. (2015) identified a relationship between higher self-reported physical activity and hearing aid use for women but not for men. Given the identification of only two studies, and noting that both studies had only basic measures of physical activity, no conclusions can be drawn relating to H2_P.

## Discussion

Investigation of the role of activity in daily-life fatigue helped address in greater depth what previous literature can inform us about the relationship between hearing loss, hearing device use and daily-life fatigue, and by association how well-being can be affected. The literature regarding the relationships between both hearing loss and hearing device use and activity is varied: there are inconsistent methodologies across studies, and only one study out of 66 mentioned power analysis (Dawes et al. 2015). Nevertheless, there is compelling evidence of activity being a potential confounding variable in investigations of listening-related fatigue. The extent to which activity might be related to listening-related fatigue depends on the type of activity, and the relationship is not always monotonic. While a change in the quantity of a given activity would likely impact the experienced fatigue, qualitative factors at the experiential level can also play a role. An individual’s well-being is likely affected by all factors in the equation, as discussed below.

### Hearing loss, hearing device use and work activity

Regarding work activity, the literature highlighted that other things being equal, people with a hearing loss are more likely to be unemployed and possibly also retired compared to people without a hearing loss. The impact of a change in work activity on fatigue is dependent on the control of, and psychology behind, the change in activity. Unemployment has been identified as potentially resulting in more long-term fatigue due to the pressures caused by aspects such as job-seeking (Lim et al. 2016). Social pressures are also present in the work place and could become a barrier to employment (Hetu et al. 1994). As an individual rarely has control over job loss and would see it as a negative, a hearing loss could result in increased fatigue due to the psychosocial difficulties involved in unemployment. Conversely, employed work itself is a potentially fatiguing activity, so a reduction in the quantity of work through unemployment might logically reduce fatigue in some individuals. Individual differences such as work demand and responsibility would likely impact the fatigue experienced during work (Åkerstedt et al. 2004). It is also the case that not all employees disclose their hearing loss to employers, which would keep work difficulty and stress high as people forgo help (Southall, Jennings, and Gagne 2011). Retirement, on the other hand, is generally associated with a reduction in fatigue, as the individual has control over the decision and views retirement as a positive. Hearing loss that results in unwanted reductions in work activity could reduce an individual’s well-being directly, as well as through subsequent increases in long term fatigue.

Results from the literature pertaining to hearing device use and work activity were equally divided. The cochlear implant studies suggested a causal relationship of hearing device affecting activity, whereas results from the hearing aid studies did not suggest a direction of effect. Cochlear implantation studies provided more support for the hypothesis than hearing aid studies, potentially due to the greater change in hearing ability post fitting. Hearing device use which results in increased work activity through a change in job status could correspond to a reduction in the fatigue caused by unemployment. Given that work activity can be fatiguing, it is therefore likely that the alleviation of fatigue from unemployment could in part be modulated by transient work-related fatigue. If, as suggested, hearing device use makes an individual more likely to find employment, stay in a job or not leave the workforce early, then socio-emotional well-being should be supported. The enjoyment/satisfaction of the job and the listening fatigue experienced during the working day are important variables that could also impact well-being.

### Hearing loss, hearing device use and social activity

The evidence of a relationship between both hearing loss and hearing device use and social activity is mixed, due in part to the insensitive measurement of social activity level, which was common in many studies. Elderly people with a hearing loss may suffer reduced social activity, which the literature suggests would offer less opportunity for the social activity itself to reduce fatigue (Oerlemans, Bakker, and Demerouti 2014; Oerlemans and Bakker 2014).Whether this is also the case for younger populations is uncertain. While the amount of social activity is important, the control and enjoyment of it is equally important as this seems to dictate the level of resulting fatigue (Ten Brummelhuis and Trougakos 2014). As previous literature has identified that hearing loss can make social situations more challenging to take part in and potentially less enjoyable (Heffernan et al. 2016; Barker, Leighton, and Ferguson 2017), it is logical to conclude that hearing loss can increase fatigue via psychological mechanisms as well as auditory ones. As such, an individual’s well-being would likely be affected by listening fatigue, sub-optimal social activity, and the interaction between the two.

Hearing aids and cochlear implants have been identified as improving quality of life (Cohen et al. 2004). A majority of evidence in this review regarding hearing device use identified an increase in social activity, which would in turn offer more opportunity to reduce fatigue, as long as the activity was enjoyed or intrinsically motivated (Ten Brummelhuis and Trougakos 2014). In addition, as hearing devices are not always beneficial in noisy social environments, the effect of a change in social activity on fatigue may be very specific to the individual. The age of participants under investigation could be an important factor. All studies which did not support H2_S used elderly population samples, in addition to only one study which did support H2_S (Fisher et al. 2015). It is possible that the behaviour of elderly people may be harder to change than for younger people, or that there is less desire for change. If this were the case, then it is possible that the “failure” of hearing devices to increase social activity in the elderly may not impact well-being, as people may be content with their current levels of activity.

### Hearing loss, hearing device use and physical activity

The hypothesis that hearing loss is related to reduced physical activity was supported by the majority of the available evidence. This could, in turn, lead to increased feelings of fatigue (Jette et al. 1996; Puetz, Flowers, and O'Connor 2008). As the experienced fatigue in question is not due to a specific situation, this example clearly demonstrates an impact on long-term (as opposed to transient) fatigue. In addition, as fatigue due to inactivity is not brought on by physical or cognitive exertion, this suggests that the fatigue may be caused by psychological mechanisms. Previous evidence indicates that both fatigue and inactivity can affect an individual’s well-being (McAuley et al. 2000; Haack and Mullington 2005), suggesting that there could be an additive effect when an individual has hearing loss. The evidence regarding hearing device use and physical activity is far too thin to draw any firm conclusions.

The relationship between both hearing loss and hearing device use and activity is a topic that has been regularly discussed in hearing loss research. However, until now there has been no assessment of the existing literature pertaining to the quantitative impact of hearing loss and hearing device use on activity, or vice versa. Some limitations of this review relate to the inclusion of certain studies whose outcome measures only partly addressed the research questions. While this was done in order to include all relevant information, it means that some findings are more reliable than others. Additionally, associations between variables should not be taken as a causal relationship.

All results involving the relationships between both hearing loss and hearing device use and activity, and the subsequent relationship with fatigue and well-being, must be taken with the understanding that the relationship is complex, with multiple factors influencing potential relationships. Hearing loss can result in a reduction in well-being due to many factors, fatigue being just one. The impact that hearing loss has on a person’s levels of fatigue and daily-life activity would be dependent on individual circumstances, and the subsequent relationship between those factors and well-being would likely be contingent on individual factors such as personality. Equally, research suggests that by fitting a hearing device the negative impact of hearing loss on an individual would be lower (Mulrow, Tuley, and Aguilar 1992). However, the use of the hearing device and satisfaction with it would be key components in any subsequent improvement in fatigue, activity or well-being.

## Conclusions

Besides the straightforward link between hearing loss and fatigue via increased effort, the extant literature suggests a potential indirect impact of hearing loss on increased fatigue (and of hearing device use on reduced fatigue) via concomitant changes in work, social or physical activity. The enjoyment and control that a person has over a given situation is likely to be a key determinant of any resulting fatigue.

Here we have demonstrated that not only is it possible that fatigue resulting from hearing loss could be directly related to well-being, but that changes in activity relating to hearing loss (and its alleviation) could impact well-being both directly and through changes in fatigue.

Given the important role of activity, knowledge of the activities undertaken by a person day-to-day is thus crucial to understanding the daily-life fatigue experienced. Therefore, future research into listening-related fatigue including measures of activity may serve to provide a better understanding of daily-life fatigue and its association to well-being.
